# Protective and Ameliorative Effects of Hydroethanolic Extract of *Piper nigrum* (L.) Stem against Antiretroviral Therapy-Induced Hepatotoxicity and Dyslipidemia in Wistar Rats

**DOI:** 10.1155/2024/5811080

**Published:** 2024-02-07

**Authors:** Doreen Enyang, Mubo A. Sonibare, Armelle D. Tchamgoue, Lauve R. Y. Tchokouaha, Fanta S. Yadang, Gael N. Nfor, Christelle W. Kom, Patrick D. H. Betote, Cedric F. Tchinda, Steveng S. K. Tiogo, Gabriel A. Agbor

**Affiliations:** ^1^Medicinal Plant Research and Drug Development Program, Pan African University Life and Earth Sciences Institute, University of Ibadan, Ibadan, Nigeria; ^2^Department of Pharmacognosy, Faculty of Pharmacy, University of Ibadan, Ibadan, Nigeria; ^3^Centre for Research on Medicinal Plants and Traditional Medicine, Institute of Medical Research and Medicinal Plants Studies, Ministry of Scientific Research and Innovations, P.O. Box 6163, Yaoundé, Cameroon; ^4^Department of Organic Chemistry, University of Yaoundé 1, Yaoundé, Cameroon

## Abstract

Antiretroviral therapy (ART) has revolutionized the lives of people living with HIV/AIDS by overall improving their quality of life and increasing life expectancy. However, ART-associated hepatotoxicity and metabolic disorders in HIV/AIDS patients are growing concerns to clinicians, especially due to the long-term use of the drugs. This study reported on the phytochemical and pharmacological profile of hydroethanolic extracts of *Piper nigrum* stem (PNS) and evaluated its protective effect against tenofovir/lamivudine/efavirenz (TLE)-induced hepatotoxicity and dyslipidemia in Wistar rats. Cytotoxic, antioxidant, and anti-inflammatory assays were performed on PNS. Thirty-six rats divided into 6 groups of 6 animals/group were administered: distilled water, 17 mg/kg TLE, 17 mg/kg TLE and 100 mg/kg silymarin, 17 mg/kg TLE, and *Piper* extract (200 mg/kg, 400 mg/kg, or 800 mg/kg) orally for 28 days. The body weight of animals was recorded every 7 days. On Day 29, the rats were sacrificed, and blood samples were collected for hematological and biochemical tests. Portions of the liver and kidneys were collected for histological evaluation, while liver homogenates were prepared from the rest to measure antioxidant enzymes. PNS possessed in vitro cytotoxic, antioxidant, and anti-inflammatory activities. A significant decrease (*p* < 0.05) in the body weight of rats treated with PNS was observed. A significant high platelet count (*p* < 0.05) was observed in the PNS800 mg/kg group. A considerable decrease in alkaline phosphatase and triglycerides was observed in the silymarin and PNS group compared to the TLE-only group. The findings also show a significant increase in catalase and glutathione in the TLE-only group compared to the normal group, while SOD decreased. Histological observations revealed normal hepatic and renal tissues in the silymarin, and PNS-treated groups compared to the normal control, while leucocyte infiltration was observed in the TLE-only group. These results suggest that PNS extract possessed antioxidant activity that alleviated TLE-induced toxicity. Further studies are necessary to understand the pharmacokinetic interactions between ART and PNS.

## 1. Introduction

The development and efficacy of antiretroviral therapy (ART) have transformed HIV infection from a death sentence to a chronic disease that is now manageable [1–3]. However, drug-induced liver injury (DILI) and metabolic disorders in immunocompromised individuals like HIV patients is becoming an increasing concern. ART-associated toxicity has emerged to be the main reason for the discontinuation or modification of ART [4, 5].

The liver is the largest and most significant organ in the human body, as it is the site of vital metabolic reactions. It detoxifies hazardous chemicals as well as synthesizes beneficial biomolecules. As a result, liver injury has serious repercussions [6]. Liver toxicity and metabolic abnormalities like dyslipidemia are most common in HIV patients [1, 7, 8], especially in sub-Saharan Africa (SSA), where there are numerous factors that may enhance the risk of hepatotoxicity [9, 10]. DILI is described as liver damage caused by any prescription or nonprescription drug, including small chemical molecules, biological agents, traditional Chinese medicines, natural medicines, health products, and dietary supplements. It is one of the most common and serious adverse drug reactions which can result in acute liver failure and possibly death, when severe [11]. DILI has become an increasingly important source of morbidity and mortality in HIV-infected people as the patients now live longer [3].

The incidence of DILI in the general population in developed countries is estimated to be between 1/100,000 and 20/100,000 people. In contrast, it is rarely recorded in SSA even though the region is a highly endemic zone. Given the high prevalence of HIV, viral hepatitis, and tuberculosis (TB) infections, as well as the well-known hepatotoxicity of their treatments, DILI is predicted to contribute to the liver disease burden of SSA [11]. Egypt, Zambia, and Eritrea are the African countries with a high liver disease death rate in the world [12]. DILI affects up to 18% of patients receiving highly active antiretroviral therapy (HAART) [13]. Adverse drug reactions due to ART were reported in 22.1% of participants in a study involving 3921 HIV-positive adults from seven teaching hospitals in Ethiopia, with 9.5% having severe forms [14]. In research conducted in Cameroon involving HIV/AIDS, TB, and HIV/TB patients who did not exhibit any sign of abnormal liver enzyme level at the time of treatment initiation, hepatotoxicity was reported after one, four, eight, and twelve weeks of follow-up with a frequency of 16.0%, 6.4%, 16.8%, and 3.2%, respectively [15].

Several mechanisms responsible for ART-associated liver toxicity in HIV patients have been reported [3, 16]. These include oxidative stress, lipotoxicity, immune-mediated injury, mitochondrial toxicity, and accumulation of toxic metabolites, with mitochondrial toxicity being the most implicated [1–5, 8, 17–19].

Tenofovir disoproxil fumarate (TDF) is one of the more tolerable and effective nucleotide reverse transcriptase inhibitors than older agents which were known for their many deleterious effects [20]. It was approved by the Food and Drug Administration in 2001 for the treatment of HIV and is now the backbone of many antiretroviral therapy combinations [20, 21]. One of these combinations is the regimen TDF300 mg/lamivudine 300 mg/efavirenz 400 mg. As a combination, observed TLE side effects are headache, nausea, nightmares, drowsiness, vomiting, insomnia, loss of appetite, diarrhea, and allergies [22].

TDF, in spite of its high level of efficacy, safety, and low metabolic side effects, has been linked to severe hepatomegaly with steatosis and severe lactic acidosis [20]. Renal and bone toxicities have also been reported especially after prolonged use of TDF [21, 23]. Renal toxicities are characterized by tubulopathies, while bone toxicity is often characterized by osteopenia, osteoporosis, osteomalacia, and bone fractures [23]. Damage to the proximal tubule by circulating plasma tenofovir after TDF hydrolysis by gut and plasma esterases is suspected to be the mechanism by which renal toxicity is induced [20]. Lamivudine, a cytidine analogue on the other hand, is known for its low toxicity at clinical doses, while neuropsychiatric adverse effects were recorded after 12 months of therapy with the efavirenz-containing regimen in HIV patients who were naïve at baseline in a Chinese cohort study [24]. These adverse events were temporary and transient. Chronic administration of efavirenz to rats resulted in distorted and disrupted kidney cytoarchitecture [25].

Cross-sectional studies across the world report the prevalence of dyslipidemia to be statistically higher in HIV patients on ART than in ART-naive HIV patients [26–29]. The present management of ART-induced toxicity is mostly based on monitoring of transaminase levels and the switching or discontinuation of ART [8, 30]. These strategies compromise the benefits of ART and make patient management challenging [31].

Medicinal plants with proven antioxidant and hepatoprotective activities like *Piper nigrum* could offer alternative treatments that are affordable, accessible, and safer [6, 32, 33]. The pharmacological activities of *Piper* species include insecticidal, antifungal, antiamoebic, antimicrobial, antiasthmatic, anti-inflammatory, antidiabetic, immunomodulatory, anticancer, hypocholesterolemic, antioxidant, analgesic, antidepressant, and neuropharmacological activities [32, 34–40]. Phytochemical screening revealed that the major bioactive compound present in *Piper* species like *Piper nigrum* is an alkaloid, piperine [41–43]. In vivo and in vitro studies have shown *Piper nigrum* to be potentially toxic causing irritations, drowsiness, indigestion, and heartburn [44]. The acute toxicity (LD50) of ethanolic extracts of *Piper nigrum* leaves revealed that the plant was not toxic to rats at concentrations of up to 5000 mg/kg [45]. Alcoholic extracts of *Piper nigrum* fruits and seeds were shown to offer protection against hepatotoxicity induced by ethionamide, para-aminosalicylic acid, and ethanol-CCl_4_ in rats [33, 46]. Furthermore, treating rats with *Piper nigrum* leaf hydroethanolic extracts reduced the effect of cafeteria diet-induced obesity [40]. Some exogenous antioxidants like Jobelyn, vitamin C, and vitamin E have been proven to potentially alleviate hepatotoxicity induced by the antiretroviral drug, nevirapine [47]. *Piper nigrum* possesses proven antioxidant activities [32, 39] which may be protective against ART-induced toxicity. In this present study, we evaluated the protective effects of *Piper nigrum* stem extract against ART-induced hepatotoxicity and dyslipidemia.

## 2. Materials and Methods

### 2.1. Plant Collection and Preparation of Crude Extract

Fresh *Piper nigrum* plants were collected at the foot of Mount Kala, in the environs of Nkolbisson, Yaoundé, Centre Region of Cameroon in January 2022. The collection followed good harvesting practices [48], with the help of a Herbarium technician and a Botanist. No authorization for the collection was required. The stems of the *Piper nigrum* stem (PNS) were separated from the leaves, dusted, cut into smaller pieces, and air-dried on mesh bags for three (3) weeks. The dried pieces of the stem were then pulverized to obtain 1.673 kg fine powder which was stored in air-tight plastic bags. The fine powder obtained was macerated for three days with ethanol/distilled water (70/30 v/v) as solvent. After filtration and evaporation of the solvent using a rotavapor (Heidolph), an extraction yield of 6.48% PNS hydroethanolic extract was obtained. This crude extract was stored in an air-tight glass container in a refrigerator at 4°C until when required.

### 2.2. Qualitative Phytochemical Screening

Phytochemical screening was performed to detect the presence of some active components in the PNS crude extract using the method of Harborne [38]. These active components were alkaloids, phenols, tannins, saponins, steroids, anthocyanins, flavonoids, and anthraquinones.

### 2.3. *In Vitro* Assays

#### 2.3.1. *In Vitro* Cytotoxic Assay

The African green monkey normal kidney Vero cell line (ATCC CRL 1586) and HepG2 cell line (85011430) (liver cancer cell lines) were used for this assay. The test samples were weighed and prepared at 1000 *μ*g/mL for extracts and 10 mM for podophyllotoxin using 100% dimethyl sulfoxide (DMSO). The method used was as described by Bowling et al. [49].

Vero cells were maintained in T-25 flasks using complete Dulbecco's modified Eagle's medium (DMEM), while Eagles' minimum essential medium (EMEM) was used to maintain HepG-2 cells. The media were supplemented with 10% fetal bovine serum, 0.2% sodium bicarbonate (w/v), 1% (v/v) penicillin-streptomycin, and 1% MEM nonessential amino acids. Cells were kept at 37°C and 5% CO_2_ with the medium renewed every 72 h. The cell density was monitored using an Etaluma 520 inverted microscope until a monolayer formed.

Confluent culture (nearly 90%) was trypsinized (0.25% Trypsin-EDTA) and centrifuged at 1800 rpm for 5 minutes, and the pellet was resuspended in the culture medium. Cells were seeded (104 cells per well) in 96-well culture plates (Costar, USA) and incubated overnight to allow cell adhesion. Following that, 10 *μ*L of serially diluted samples was added in duplicate to plate wells. For 48 hours, the plates were incubated at 37°C and 5% CO_2_ atmosphere.

As a positive control, 10 *μ*M podophyllotoxin was added, and wells containing untreated cells were included as a 100% growth control. A volume of 10 *μ*L of resazurin stock solution (0.15 mg/mL in sterile PBS) was added to each well and incubated for an additional 4 hours. Fluorescence was then measured with a Magelan Infinite M200 fluorescence multiwell plate reader (Tecan) at 530 nm and 590 nm for excitation and emission, respectively. The percentage of cell viability was calculated in relation to the negative control, and the concentration that reduced 50% of cell viability (CC50) was determined by nonlinear regression using the GraphPad Prism 8.0.2 software (San Diego, California).

#### 2.3.2. *In Vitro* Antioxidant Assays

The PNS extract was prepared at a stock concentration of 1000 *µ*g/mL. Five assays were used to assess the antioxidant activity of the extract.

*(1) 1,1-Diphenyl-2-picrylhydrazyl (DPPH) Radical Scavenging Activity*. The DPPH free radical scavenging activity of the PNS extract was measured using the Sanchez-Moreno et al. method [50], with some modifications. In brief, 1.95 mL of a DPPH methanolic solution was mixed with 0.05 mL of the PNS extract at various concentrations (100–1000 *μ*g/mL) (0.1 mM). The mixture was homogenized and incubated in the dark for 30 minutes at room temperature. Methanol was used in place of the extract in the control tube. Ascorbic acid at various concentrations (10–100 g/mL) was used as a reference. The experiment was carried out in triplicate, and the absorbance was measured at 517 nm. The free radical scavenging activity was calculated as a decrease in DPPH absorbance using the following equation:

(1) $$\begin{array}{c} Inhibition\;percentage\;of\;PNS\;on\;DPPH\;\left(\%\right)=\frac{\left(A0-A1\right)}{A1}\;x\;100, \end{array}$$

where *A*0 represented the absorbance of the control reaction and *A*1 represented the absorbance of the PNS sample extract.

*(2) 2,2′-Azinobis-(3-ethylbenzothiazolin-6-sulfonic Acid) (ABTS) Scavenging Activity*. Each reaction tube contained 1 mL of diluted ABTS solution and 10 *μ*L of the extract at varying concentrations (0 to 1 mg/mL). The tubes were agitated and incubated at room temperature in the dark for 15 minutes. The absorbance was measured at 734 nm. Ascorbic acid (100 *μ*g/mL) was used as a standard. The experiment was performed in triplicate. The percentage inhibition of PNS on ABTS was calculated according to the following equation:

(2) $$\begin{array}{c} Inhibition\;percentage\;\left(\%\right)=\frac{\left(Abs\;ABTS\;-\;Abs\text{ extract}\right)}{Abs\;ABTS}\;x\;100, \end{array}$$

where Abs ABTS is the absorbance of ABTS and Abs extract is the absorbance of PNS extract.

The method used was that described by Re et al. with some modifications [51].

*(3) Ferric Reducing Antioxidant Power (FRAP) Assay*. The reducing power of iron (Fe3+) of PNS extract was determined using the method of Oyaizu [52] and performed in triplicate. 2.5 mL of 0.2 M phosphate buffer solution (pH 6.6) and 2.5 mL of 1% potassium ferricyanide (K_3_Fe(CN)_6_) solution were mixed with 1 mL of the extract at varying concentrations (100–1000 *μ*g/mL). This mixture was incubated in a water bath at 50°C for 20 minutes, and 2.5 mL of 10% trichloroacetic acid was added to stop the reaction. The tubes were centrifuged at 3000 rpm for 10 min, and the supernatant was collected. To 2.5 mL of each supernatant were added 2.5 mL of distilled water and 0.5 mL of an aqueous solution of iron chloride III (0.1% FeCl_3_). Distilled water replaced the extract which served as the blank, and 100 *μ*g/mL ascorbic acid served as the standard. The absorbance (Abs) was read at 700 nm. An increase in absorbance corresponds to an increase in the reducing power of the extracts tested. The FRAP value of the test samples was calculated using the following equation:

(3) $$\begin{array}{c} FRAP\;value=\frac{\left(Abs\;sample\;-\;Abs\;blank\right)}{\left(Abs\;standard\;-\;Abs\;blank\right)}\;x\;concentration\;of\;standard. \end{array}$$

The results were expressed in milligrams of ascorbic acid equivalent per gram of dry extract (*µ*g EAA/mg extract).

**Figure 1:**
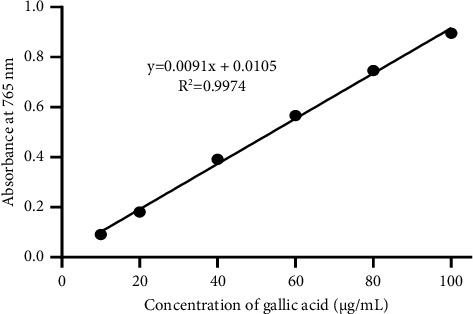
The standard curve of absorbance of gallic acid vs. concentration of gallic acid.

*(4) Total Phenolic Content Assay*. The total phenolic content of PNS was assessed using the Folin–Ciocalteu reagent based on the method described by Li et al. [53] with few modifications. 0.5 mL of Folin–Ciocalteu reagent (2  diluted 10 times) was mixed with 100 *μ*L of 1000 *μ*g/mL PNS extract. After 4 minutes of incubation at room temperature, 400 *μ*L of 7.5% calcium carbonate (CaCO_3_) was added, and the mixture agitated and was left at room temperature for 2 hours. A gallic acid stock solution of 100 *μ*g/mL was prepared and used as the standard. From this stock solution, the absorbance of six different concentrations (10, 20, 40, 60, 80, and 100 *μ*g/mL) was measured at 765 nm and converted to phenolic contents to obtain a standard calibration curve of gallic acid. Distilled water was used as the blank. Each experiment was performed in triplicate. The total phenolic content of the PNS extract was calculated using the calibration curve of gallic acid (Figure 1) and expressed as mg equivalent of gallic acid per gram of extract (mg EGA/g extract).

From the equation of the line,

(4) $$\begin{array}{c} Y=0.0091x+0.0105,\\ R2=0.9974. \end{array}$$

The phenolic content of PNS was calculated using the following equation:

(5) $$\begin{array}{c} Total\;phenolic\;content\left(\frac{\text{mgEGA}}{g}\text{extract}\right)=\frac{∆OD}{a\times m}, \end{array}$$

where ΔOD = optical density sample – optical density blank, *x* is the slope of the standard curve, and *m* is the mass of extract (g).

*(5) The Total Antioxidant Capacity (TAC) Assay*. The phosphomolybdenum method as described by Prieto et al. [54] was used to measure the total antioxidant capacity of PNS. The reaction tube consisted of 0.2 mL of extract at different concentrations (100 *μ*g/mL to 1000 *μ*g/mL) and 2 mL of working reagent solution (0.6 M H_2_SO_4_ 98%, 28 mM NaH_2_PO_4_, and 4 mM ammonium molybdate). The tubes (in triplicate) were shaken and incubated at 95°C for 90 min in a water bath. After cooling to room temperature, the absorbance (abs) of the solutions was measured at 765 nm against a blank (2 mL of the reagent solution and 0.2 mL of distilled water). Ascorbic acid at 100 *μ*g/mL was used as a standard. The total antioxidant capacity which was expressed in *μ*g equivalent of ascorbic acid per gram of extract (*μ*g EAA/mg extract) was calculated using the following equation:

(6) $$\begin{array}{c} TAC\;value\left(\frac{\text{EAA}}{\text{mg extract}}\right)=\frac{\left(Abs\;sample-Abs\;blank\right)}{\left(Abs\;standard-Abs\;blank\right)}\times concentration\;of\;standard. \end{array}$$

#### 2.3.3. *In Vitro *Anti-Inflammatory Assays

*(1) Effect of PNS on Protein Denaturation*. Using a method adopted from Gunathilake et al. [55], each reaction tube held 100 *μ*L of a test sample (PNS or diclofenac) of different concentrations (50 *μ*g/mL to 1000 *μ*g/mL) and 0.5 mL of 5% bovine serum albumin (BSA) prepared in 100 mL phosphate buffer saline (PBS) at pH 7.4. A mixture of 0.5 mL BSA and 1.1 mL PBS was used as a control. Each tube was shaken and incubated at 37°C for 15 min. After incubation, the tubes were put in a water bath at 70°C for 10 min. After cooling, the resulting solution was very viscous. A volume of 1 mL of distilled water was added to each tube, and the optical density (O.D) was read at 660 nm after centrifugation at 3000 rpm. The percentage inhibition on protein denaturation was calculated using the following equation:

(7) $$\begin{array}{c} Denaturation\;inhibition\;percentage\;\left(\%\right)=\frac{\left(O.D\;control\;-\;O.D\mathrm{sample}\right)}{O.D\;control}\;x\;100. \end{array}$$

*(2) Proteinase Inhibitory Activity*. The method used was that described by Sakat et al. with some modifications [56]. The reaction mixture which had a final volume of 2.5 mL was made up of 0.5 mL test sample (extract or diclofenac) at different concentrations, 0.5 mL of a solution of 60 *μ*g of trypsin in 100 mL of 20 mM Tris-HCl buffer at pH 7.4, 0.5 mL of 0.8% casein, and 1 mL of 70% perchloric acid. In the place of the test sample, 0.5 mL distilled water was used as the control. The percentage inhibition was calculated using the following equation:

(8) $$\begin{array}{c} Inhibition\;percentage\;\left(\%\right)=\frac{\left(O.D\;control\;-\;O.D\;sample\right)}{O.D\;control}\;x\;100, \end{array}$$

where O.D is the optical density.

### 2.4. *In Vivo* Studies

#### 2.4.1. Ethical Considerations

The in vivo part of this study was conducted in compliance with the practices and principles of the Institute for Laboratory Animal Research Division on Earth and Life Studies, respecting the 2011 Guide for the Care and Use of Laboratory Animals, 8th edition [57]. The study proposal received a favorable opinion from the Joint Institutional Review Board for Animal and Human Bioethics, University of Yaoundé 1, Yaoundé, Cameroon. An official document to obtain and use the antiretroviral therapy regimen, tenofovir 300 mg/lamivudine 300 mg/efavirenz 400 mg (TLE), was issued by the Cameroon National AIDS Control Committee.

#### 2.4.2. Experimental Animals and Treatment

The experimental animals consisted of thirty-six male Wistar rats, 5 to 6 weeks old weighing 92–138 g, purchased from the Animal House of the Laboratory of Animal Physiology, University of Yaoundé 1, Yaoundé, Cameroon, and housed in the animal facility of the Centre for Research on Medicinal Plants and Traditional Medicine, Institute of Medical Research and Medicinal Plant Studies, Yaoundé, Cameroon. They were subjected to 12 hours of light and 12 hours of darkness, a temperature of 25 ± 3°C, and had access to standard rat food and water. The animal facility was in a cool and quiet environment away from noise. After 10 days of acclimatization, the rats were divided into six groups (*n* = 6) based on their body weight.

The calculation of the dose of TLE for groups 2 to 6 was performed following the methods of Umar et al. [48] and Oghenesuvwe et al. [58] by direct calculation from human dose as follows.

*(1) Calculation of Dose of TLE (for Groups 2 to 6) by Direct Calculation from Human Dose*. A human (at least 35 kg) requires 300 mg of tenofovir in the tenofovir 300 mg/lamivudine 300 mg/Efavirenz 400 mg regimen per day.

Therefore, the dose required for an animal having an average weight of 100 g was calculated using the following equation:

(9) $$\begin{array}{c} \mathrm{Dose}\;\mathrm{of}\;\mathrm{TLE}\;\mathrm{for}\;a\;100\;g\text{ animal}=\left(\frac{\text{dose of human}}{\text{body weight of human}}\right)\times \text{body weight of animal}\\ =\left(\frac{300\text{ mg}}{35000\;g}\right)\times 100\;g=0.857\text{ mg}. \end{array}$$

NB: The dose was doubled to ensure hepatotoxicity can be induced within the 28-day test period.

Therefore, the dose administered to test Wistar rats = 0.857 × 2 = 1.714 mg.

This 1.714 mg is per 100 g, which implies 17.14 mg per 1000 g(a kg).

1.714 mg was prepared in 0.5 mL of distilled water for one rat.

For 6 rats, 10.284 mg was dissolved in 3 mL of distilled water to be administered in a day.

The Wistar rats received orally the following products daily for 28 days as follows:

  Group 1: normal control administered 1 mL distilled water
  Group 2: negative control administered 1 mL of 17 mg/kg TLE
  Group 3: positive control administered 0.5 mL of 17 mg/kg TLE + 0.5 mL of 100 mg/kg silymarin
  Group 4: PNS200 test group administered 0.5 mL of 17 mg/kg TLE + 0.5 mL of 200 mg/kg PNS extract
  Group 5: PNS400 test group administered 0.5 mL of 17 mg/kg TLE + 0.5 mL of 400 mg/kg PNS extract
  Group 6: PNS800 test group administered 0.5 mL of 17 mg/kg TLE + 0.5 mL of 800 mg/kg PNS extract

All solutions were prepared using distilled water.

#### 2.4.3. Sacrifice of Rats and Sample Collection

After the 28-day treatment period, the rats were anesthetized in ether. Blood was collected into EDTA tubes by cardiac puncture and centrifuged at 3000 rpm for 15 min at 4°C. The plasma collected was stored in Eppendorf tubes at −20°C for further evaluation of hematological and biochemical parameters. Two rats from the TLE + PNS800 mg died before the end of the 28-day test period.

Each rat was dissected to obtain the liver and kidneys. The organs were rinsed in 1.15% potassium chloride (KCl) solution, dried with a paper towel, and weighed. Using a portion of the liver and an ice-cold Tris-HCl 50 mM solution, 10% w/v homogenate was each prepared and centrifuged at 3000 rpm for 25 minutes at 4°C. The resulting supernatant was used to assay the antioxidant enzymes (catalase, superoxide dismutase, and reduced glutathione). The remainder of the liver and kidneys were preserved in 10% formalin for histological analysis.

#### 2.4.4. Determination of Body Weight Evolution and Relative Mass of Organs

Body weight of each animal was taken at the beginning of each week. The variation in weekly body weight was calculated using the following equation:

(10) $$\begin{array}{c} \text{body weight evolution }\left(\%\right)=\left(W\;-\mathrm{Wi}\right)\times 100, \end{array}$$

where *W* represented the weight of the animal at the start of the week in grams and *Wi* represented the weight of the animal before experimentation in grams.

The relative mass of the organs (liver and kidneys), RMO, was calculated using the following equation:

(11) $$\begin{array}{c} RMO\;\left(\%\right)=\frac{\mathrm{mass}\text{ of organ }\left(g\right)}{\mathrm{mass}\text{ of animal }\left(g\right)}\times 100. \end{array}$$

#### 2.4.5. Hematological Parameter Analyses

The blood samples for hematological analyses were collected in EDTA tubes dipped in ice-cold water and immediately transported to the laboratory for analysis. White blood cells, lymphocytes, monocytes, neutrophils, eosinophils, platelets, red blood cells, hemoglobin, hematocrit, mean cell volume, mean cell hemoglobin, and mean cell hemoglobin concentration were all measured. These tests were performed at the Hematology Laboratory of the Central Hospital of Yaoundé, Yaoundé, Cameroon.

#### 2.4.6. Biochemical Parameter Analyses

The concentration of transaminases (alanine aminotransferase and aspartate aminotransferase), alkaline phosphatase, creatinine, triglycerides, total cholesterol, and total proteins in the plasma was measured through colorimetric tests using commercial kits according to the manufacturers' procedures.

#### 2.4.7. Antioxidant Enzyme Analyses

Three antioxidant activity assays were evaluated using the liver homogenate. The catalase activity was assessed using the method of Sinha [59], superoxide dismutase was assessed using the method of Misra and Fridovich [60], and reduced glutathione was assessed using the method of Ellman [61].

#### 2.4.8. Histological Analysis

The kidneys and liver collected from the Wistar rats were fixed in Bouin's fluid, dehydrated, and embedded in paraffin. Using a Reichert-Jung 2030 microtome, the paraffin blocks containing the organs were cut to a thickness of 5 *μ*m and stained with hematoxylin and eosin. They were assembled and observed under the microscope (Scientico STM-50) equipped with a Celestron 44421 digital camera connected to a Toshiba Tecra A9 computer. The Digital Microscope Suit 2.0 software was used to take the photomicrographs. These analyses were performed at the Laboratory of Animal Physiology, University of Yaoundé 1, Yaoundé, Cameroon.

### 2.5. Statistical Analyses

The results were calculated using Microsoft Excel 365 and expressed in mean ± standard deviation (for in vitro assay results) or mean ± standard error of the mean (for in vivo assay results). GraphPad Prism 8.0.2 software was used for data statistical analysis. The values obtained were compared using one-way ANOVA followed by Tukey multiple comparison posttest. *p* values less than 0.05 were considered statistically different.

## 3. Results and Discussion

The liver plays a crucial role in normal homeostasis and the metabolism of drugs [62]. Given that HIV and its comorbidities are highly widespread in sub-Saharan Africa and that DILI can have a variety of causes, the condition is becoming a growing concern among HIV patients taking HAART [9]. Limited data on the incidence and prevalence of this public health issue exist, with few studies carried out to seek novel strategies for the effective management of patients [9, 10]. This study demonstrated the antioxidant and anti-inflammatory properties of *Piper nigrum* stem as supported by earlier studies [32, 43]. It is the first study to evaluate the role of *Piper nigrum* hydroethanolic extract in TLE-induced hepatotoxicity and dyslipidemia in Wistar rats.

### 3.1. Phytochemical Screening

The phytochemical screening results revealed the presence of bioactive components such as alkaloids, polyphenols, flavonoids, triterpenes, steroids, anthocyanins, saponins, and tannins in the PNS extract. Anthraquinones were however absent in this extract. The results are like those of Kighiga and Kalunta who demonstrated the presence of tannins, flavonoids, and alkaloids in the aqueous extract of *Piper nigrum* [36]. The use of a plant in the management and treatment of various diseases is significantly influenced by the bioactive components it contains.

### 3.2. *In Vitro* Cytotoxic Activity of PNS

**Figure 2:**
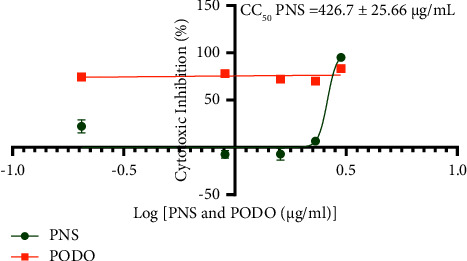
Cytotoxic activity of PNS on HepG2 cell lines.

**Figure 3:**
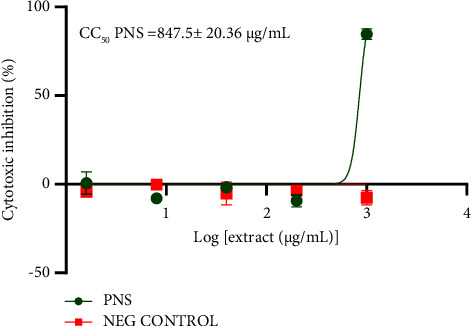
Cytotoxic activity of PNS on Vero cell lines.

The cytotoxic concentration 50% (CC50) of PNS was 847.5 ± 20.36 *μ*g/mL for Vero cells and 426.7 ± 25.66 *μ*g/mL for HepG2 cells. This means that PNS is safer to the normal kidney cell than to the cancerous liver cell. PNS was cytotoxic to both the normal kidney (Vero) cells and liver cancer (HepG2) cell lines at a concentration of 1 mg/mL as shown in Figures 2 and 3. These results are backed by Wang and collaborators [35], who concluded that *Piper* extract possesses anticancer properties. Cytotoxicity of a plant is a crucial factor to be considered before conducting an in vivo assay. Plants with low toxicity to host cells are thought to be potential candidates for new drug development, as they will not destroy the healthy living cells of the host, while destroying cancer cells [63]. The cytotoxic property of PNS could be due to the presence of compounds like piperine and piperlongumine. Piperlongumines target p38 signaling selectively destroying cancer cells and causing autophagy. Piperine triggers cell cycle arrest as well as autophagy [35].

### 3.3. Antioxidant Activity of PNS

This study revealed yet again the antioxidant property of the *Piper nigrum* plant as earlier proven by Agbor et al. [32] and Redina et al. [34]. The inhibitory concentration 50% (IC50) of PNS on DPPH and ABTS radicals was 1.39 ± 0.05 mg/mL and 0.97 ± 0.07 mg/mL, respectively. The FRAP value which was concentration-dependent was 107.77 ± 2.13 *μ*g/mL equivalent to ascorbic acid, at a concentration of 1.0 mg/mL of PNS. Meanwhile, TAC of PNS at a 1.0 mg/mL was 79.06 ± 1.92 *μ*g equivalent of ascorbic acid. The total phenolic content of the extract which was 965.01 *μ*g equivalent of gallic acid/mg was, however, less than the 9.8–15.9 mg catechin equivalent/g of the three *Piper* species reported by Agbor and colleagues [32]. The antioxidant property of *P. nigrum* is largely due to the flavonoids and phenols present in the extracts which protect the body against free radical damage [38].

### 3.4. Anti-Inflammatory Activity of PNS

The effect of the test extract on the two in in vitro anti-inflammatory models demonstrated that the PNS extract exhibited a considerable inhibitory effect on BSA denaturation and proteinase activity. The percentage inhibition of PNS on BSA denaturation was from 62.88% to 74.43% within the concentration range of 0.1 mg/mL to 1.0 mg/mL, while its antiproteinase activity was between 44.08% and 93.54% for the same range of concentrations of the extract. The results are in accordance with studies that reported piperine as the major alkaloid found in *P. nigrum* with potent analgesic and anti-inflammatory properties [43, 64]. The *Piper* genus constitutes one major class of medicinal plants used to manage pain and inflammatory disorders in folkloric practice [65].

### 3.5. Effect of the 17 mg/kg TLE Regimen and PNS Extract on the Weight Evolution of the Wistar Rats

**Figure 4:**
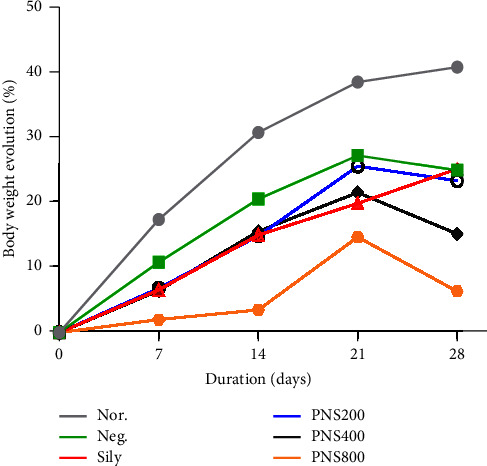
Effect of the 17 mg/kg TLE regimen and PNS extract on the body weight evolution of the Wistar rats.

Figure 4 presents the evolution of the body weight of the experimental Wistar rats in each group during the 4 weeks of treatment with TLE, silymarin, and the PNS extracts. An increase in body weight was observed in all the groups with the animals in the normal group gaining weight the most throughout the experiment (40.99%).

On day 7, PNS800 inhibited the increase in body weight of 2.0% (*p*=0.005) compared to the normal group. At the end of the second week of treatment (Day 14), PNS200 (*p*=0.04) and PNS800 (*p*=0.0003) inhibited the body weight gain compared to that of the normal group. PNS800 also inhibited the increase in body weight compared to that of the negative group (*p*=0.04). On day 21, PNS800 significantly inhibited body weight increase (*p*=0.015). In the last week of treatment, PNS200, PNS400, and PNS800 caused a significant inhibition in weight gained by 23.4% (*p*=0.046). 15.24% (*p*=0.0013), and 6.38% (*p* < 0.0001), respectively.

This result is supported by the fact that ART is associated with lipoatrophy (loss of fat tissues) [66]. NRTIs and PIs have been demonstrated to impair adipocyte function, lipid metabolism, and glucose metabolism. These drugs inhibit adipocyte differentiation as well as adiponectin expression, secretion, and release from adipose tissues [66, 67]. Adiponectin plays an important role in glucose regulation and fatty acid oxidation [67]. This is indicative that antiretroviral drugs could have an impact on body fat distribution and lipid metabolism [68]. PNS at 200, 400, and 800 mg/kg doses caused a decrease in body weight in test groups compared to the negative control group, further inhibiting weight gain. The thermogenic effect of *P. nigrum* and its action on fat metabolism may be responsible for the additional weight loss [40]. The potent alkaloid, piperine, inhibits lipid accumulation by activating metabolic enzymes such as lipoprotein lipase and adenosine monophosphate-activated protein kinase (AMPK), which promotes lipid use by peripheral tissues [69]. It also contains phenolic compounds and flavonoids, both of which aid in thermogenesis and reduce the expression of genes involved in weight gain and preadipocyte differentiation into adipocytes [40].

### 3.6. Effect of the 17 mg/kg TLE Regimen and PNS Extract on the Relative Weight of the Kidney and Liver

**Figure 5:**
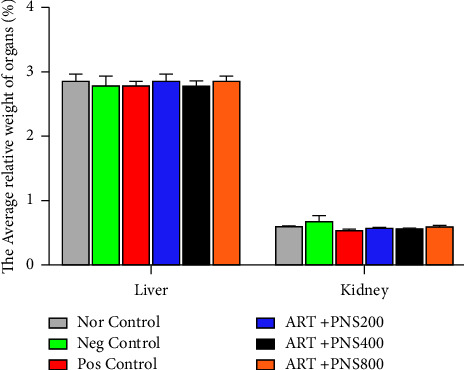
Effect of the 17 mg/kg TLE regimen and PNS extract on the relative weight of the kidneys and liver of the Wistar rats. Values are expressed as mean ± SEM, *n* = 6. Nor = administered distilled water; Neg = TLE only; Pos = TLE + silymarin; TLE + PNS 200 mg; TLE + PNS 400 mg; TLE + PNS 800 mg.

The effects of the treatment on the relative weight of the kidneys and livers in each group are presented in Figure 5. Although the average relative weights of the liver were similar in all groups (*p*=0.93), the 17 mg/kg TLE dose regimen nonsignificantly increased the relative weights of the kidney in the negative control group compared to the other groups (*p*=0.14). This result corroborates with that of a study which demonstrated that the administration of antiretroviral drugs in Wistar rats could cause an increase in the weight of their kidney especially at higher doses [70]. The *P. nigrum* dose-dependent extracts further caused a decrease in the relative weights of the kidneys in test groups compared to the negative control groups. This may be due to the fact that *P. nigrum* triggers fat metabolism [40]. There is evidence of a substantial relationship between kidney dimensions and renal function with prior research suggesting that kidney dimension measurements could be essential in the appraisal of a renal function [71, 72]. A group of patients with smaller kidney sizes had the highest creatinine level and lower estimated glomerular filtrate compared to the group with bigger kidney sizes [72]. As such, higher doses of PNS extract could be detrimental to the health of the kidney.

### 3.7. Effect of the 17 mg/kg TLE Regimen and PNS Extract on the Hematological Profile

As presented in Table 1, hematological parameters were not altered in the treated groups compared to the normal control group except for the platelet count (*p*=0.004). ART has been associated with hematological toxicity defined as thrombocytosis. This implies that patients on HAART have a risk of developing cardiovascular diseases [73]. PNS-treated rats had less platelet counts than the group treated with TLE only except the group treated with 800 mg of the extract. Our results are supported by those of Emon et al. [74], who from their research demonstrated that methanolic extracts of *P. nigrum* are capable of inhibiting coagulation, which is an important component of thrombolytic management that does not interfere with the body's normal clotting process. The very high platelet value observed in the PNS800 group could be because the dose of PNS is getting towards a toxic dose.

### 3.8. Effect of 17 mg/kg TLE Regimen and PNS Extract on the Biochemical Profiles

ALT, AST, ALP, and TP are reliable indicators for hepatotoxicity. The results as presented in Table 2 show an insignificant increase in the level of ALT and AST in the TLE-only treated group compared to the normal control. The very high level of ALT (90.82 U/L) in the group treated with 200 mg of the extract may be due to idiosyncratic reactions. A mechanism by which HAART induces liver injury is inhibiting the multienzyme complex, cytochrome P450 (CYP450). CYP450 enzymes are responsible for the metabolism of xenobiotics. NRTIs such as tenofovir and lamivudine, which are part of the TLE regimen, have been reported to cause mitochondrial injury. NRTIs increase the lipid content of the cell membrane causing stress to the endoplasmic reticulum, as a result, mitochondrial dysfunction [3, 62]. PNS extracts at different concentrations maintained the levels of AST in the treated group compared to the TLE-only control group. These results are like those of Zodape and Gaikwad [33], who showed that ethanolic extracts of *P. nigrum* seeds reduced AST levels in ethionamide and p-amino salicylic acid-intoxicated Sprague-Dawley rats, thereby preventing hepatotoxicity. This is likely possible due to the antioxidants present in *P. nigrum* which scavenge free radicals [33, 75].

The significantly high level of total proteins (TP) and triglycerides in the negative control may indicate a higher degree of liver injury caused by the TLE regimen. This result is as those of Adaramoye et al. [70] wherein the antiretroviral drug nevirapine caused a considerable increase TP in the nevirapine-treated groups different from the normal. *Piper nigrum* and silymarin were responsible for lowering TP levels as seen in the result of the positive control group. Our results are like those of Mballa et al. [40], who demonstrated that *P. nigrum* kept total proteins towards normal levels proving that the plant has hepatoprotective activity due to its bioactive compounds with antioxidant properties. It should be emphasized, however, that ALT and AST levels are generally increased in situations of injury to other organs, such as the heart [76]. The total protein value can be used to distinguish between hepatic injury and harm to other organs as the bulk of plasma proteins is produced in the liver [47].

The increase in triglycerides in the negative control group supports the fact that ART increases lipolysis while suppressing lipogenesis, resulting in decreased free fatty acid uptake by adipocytes, and stored triglycerides are increasingly released into the bloodstream [67]. Dave and collaborators concluded from their studies in South Africa involving HIV-infected patients that triglycerides, total cholesterol, and low-density lipoprotein cholesterol are frequently elevated in patients receiving ART. Protease inhibitors (PIs), stavudine and zidovudine, and the nonnucleoside reverse transcriptase inhibitor (NNRTI), efavirenz, are antiretroviral medications linked to the development of dyslipidemia; therefore, participants on ART had higher triglycerides, TC, LDLC, and HDLC than participants who had never received ART [77]. Rats given silymarin and *P. nigrum* stem extract showed general protective effects against high TG levels, as was the case of the study of Mballa and colleagues [40]. This could suggest that our extract significantly lowered TGs. The mechanism involved has been associated with piperine which inhibits lipid and lipoprotein accumulation by modulating lipid metabolism enzymes such as lecithin-cholesterol acyl transferase and lipoprotein lipase [78]. Once again, this has proven the beneficial effect of *P. nigrum* in the management of dyslipidemia and other metabolic disorders which may arise from antiretroviral therapy.

### 3.9. Effect of the of 17 mg/kg TLE Regimen and PNS Extract on the Antioxidant Enzymes in the Liver

**Figure 6:**
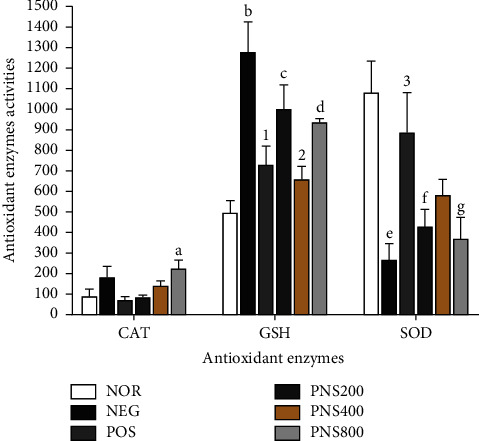
Effect of the 17 mg/kg TLE and PNS extract on the antioxidant enzymes in the liver. Each bar represents mean ± SEM, *n* = 6. Nor = administered distilled water; Neg = ART only; Pos = ART + silymarin; ART + PNS 200 mg; ART + PNS 400 mg; ART + PNS 800 mg; a, b, c, d, e, f, and g represent significant differences with respect to the normal group in each assay, while 1, 2, and 3 represent a significant difference with respect to the negative control group. CAT was measured in mM of H_2_O_2_/ming of organ, GSH in mol/g of organ, and SOD in unit/mg of protein. CAT = catalase; SOD = superoxide dismutase; GSH = reduced glutathione.

The TLE regimen caused a significant increase in catalase and reduced glutathione in the negative control group compared to the normal group while significantly reducing superoxide dismutase as presented in Figure 6. These research findings agree in part with those of Quaye et al. who reported that there was a significant increase in the antioxidant enzymes, catalase, and GSH activities in patients treated with ART compared to ART-naïve HIV patients and healthy individuals [79]. The increase in catalase and GSH mean-specific activities was likely an adaptive reaction to the oxidative challenge caused by the drug as reported by Olaniyan et al. who concluded that antiretroviral drugs like lamivudine induce liver toxicity in rats which are mediated by oxidative stress [80]. HAART, especially NTRIs, causes oxidative stress due to the production of reactive oxygen species (ROS) [3]. The increase in catalase and GSH observed could be due to scavenging of the ROS that are produced. These results are, however, different from the research that showed a significant reduction in the activities of GSH and catalase when 8-week-old male Wistar rats were treated with 50 mg/kg tenofovir for 4 weeks [81]. The antioxidants present in PNS considerably reduced catalase and GSH and increased SOD towards normal. These results corroborate the research that proved that antioxidants like vitamin E, vitamin C, and Jobelyn can modulate tenofovir and nevirapine-induced hepatotoxicity in rats [39, 81].

### 3.10. Effect of the 17 mg/kg TLE Regimen and PNS Extract on the Histology of the Kidneys and Livers

**Figure 7:**
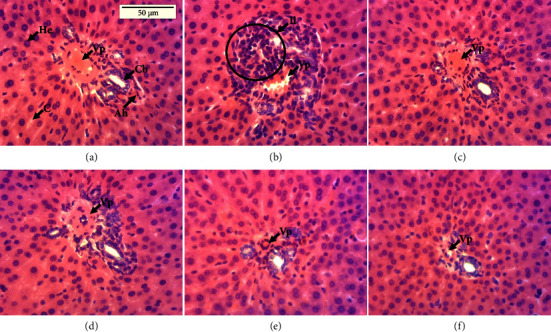
Photomicrographs of the liver (X250); hematoxylin-eosin staining. (a) Normal control; (b) negative control batch; (c) positive control; (d–f) groups receiving the extract at the respective doses of 200, 400, and 800 mg/kg; Vp = hepatic portal vein; He = hepatocyte; Cs = capillary sinusoid; Cb = bile canaliculus; Il = leukocyte infiltration.

Histological analysis of the liver (Figure 7) revealed the normal control, healthy hepatic tissue showing the portal space (portal vein, hepatic artery, and bile canaliculus) and spans of hepatocytes separated from each other by sinusoidal capillaries. Compared to normal animals, histopathological changes marked by the presence of leukocyte infiltrations were observed in the animals of the negative control group. In the group treated with the reference substance (silymarin), as well as those that received the extract at different doses, a reorganization of the liver towards that of the normal control group was observed.

**Figure 8:**
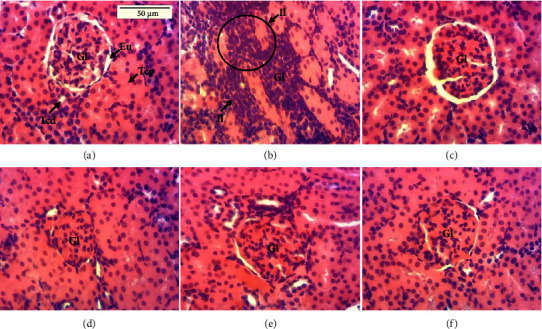
Photomicrographs of the kidney (X250); hematoxylin-eosin staining. (a) Normal control; (b) negative control; (c) positive control; (d–f) groups receiving the extract at the respective doses of 200, 400, and 800 mg/kg; Gl = glomerulus; Eu = urinary space; Tcd = distal convoluted tubule; Tcp = proximal convoluted tubule; Il = leukocyte infiltration.

Photomicrographs of the longitudinal section of the kidney (Figure 8) showed the normal control, normal renal parenchyma (glomerulus, urinary space, and distal and proximal-convoluted tubules were observed). In comparison to the normal control, histopathological changes marked by leukocyte infiltrations were observed in the negative control. Kidney tissues of the rats administered the PNS extracts and the reference drug silymarin were similar in appearance to the normal control.

From the photomicrographs of the liver and kidney of the different treatment groups, leukocyte infiltrations were observed in the group intoxicated with TLE compared to the normal or PNS-treated groups. These results are in accordance with earlier research which clearly showed that tenofovir induces renal damage in Wistar rats [82, 83]. Also, they are likened to the research in which nevirapine severely altered the architecture of the liver, resulting in significant necrosis and severe portal and central venous congestion [70]. Tenofovir is specifically toxic to the proximal convoluted tubules and proximal tubular mitochondria [83]. *Piper nigrum* extracts and the silymarin standard prevented hepatic and renal architectural damage leading to reorganization of the organs. These findings agree with that of Zodape and Gaikwad, who revealed that *Piper nigrum* seed extracts rearranged and regenerated healthy hepatic cells in rats intoxicated with ethionamide and para-aminosalicylic acid [33]. However, combining complementary and alternative medicine (CAM) with ART may result in more clinically significant pharmacokinetic (PK) interactions, especially between CAMs and protease inhibitors (PIs) and nonnucleoside reverse transcriptase inhibitors (NNRTIs), which can alter how drug efflux transporters, like P-glycoprotein (P-gp) and/or cytochrome P450 isoenzymes, like CYP3A4, intervene in the absorption and elimination of drugs in the small intestines and liver [84, 85]. Thus, despite the significant benefits of medicinal plants in the management of HIV comorbidities, extensive *in vitro* testing of herbal remedies for interference with CYP isoenzyme and P-gp activity on pharmacokinetics, PK, of ART are necessary [85].

## 4. Conclusion

This study evaluated the phytochemical and *in vitro* pharmacological profile of 70% hydroethanolic extracts of the stem of *Piper nigrum* stem (PNS). *In vivo* assays using 36 Wistar rats were further conducted using the extract to ascertain its protective effects against hepatotoxicity and dyslipidemia induced by the antiretroviral regimen TLE for 28 days. From the results of *in vitro* assays, it was shown that the PNS extract possessed active components such as alkaloids, flavonoids, and polyphenols, which are responsible for its cytotoxic, antioxidant, and anti-inflammatory properties. The findings from the *in vivo* tests showed that PNS could ameliorate hepatotoxicity and dyslipidemia induced by the TLE regimen by regulating hepatic and lipid functions towards normal. Also, the antioxidants present in the plant can scavenge free radicals which cause oxidative stress to the kidney and liver, thereby regenerating healthy hepatic and renal cells. Medicinal plants with proven antioxidant and hepatoprotective activities like *Piper nigrum* could offer alternative treatments that are affordable and accessible.

The mechanism by which *Piper nigrum* elicits its hepatoprotective activity has not been fully understood. In subsequent studies, we intend to isolate the specific active components responsible for its hepatoprotective activity and assess the molecular mechanisms of these active compounds and extracts of *Piper nigrum* on key proteins involved in ART-induced hepatotoxicity which could be essential targets in the design and development of new therapeutic molecules.

## Figures and Tables

**Table 1:** Effect of the 17 mg/kg TLE regimen and PNS extract on the hematological profile.

	Normal	Neg. control	Positive control	ART + PNS200	ART + PNS400	ART + PNS800
White blood cells (10^3^/*μ*L)	12.60 ± 2.78	11.50 ± 0.95	13.57 ± 1.14	11.77 ± 1.54	10.05 ± 1.45	8.53 ± 1.83
Lymphocytes (10^3^/*μ*L)	9.60 ± 2.48	8.60 ± 0.82	9.47 ± 0.90	8.66 ± 1.14	7.79 ± 1.09	6.43 ± 1.55
Monocytes (10^3^/*μ*L)	0.53 ± 0.34	0.17 ± 0.01	0.66 ± 0.27	0.21 ± 0.07	0.15 ± 0.03	0.11 ± 0.04
Neutrophils (10^3^/*μ*L)	2.36 ± 0.19	2.59 ± 0.32	3.30 ± 0.49	2.75 ± 0.37	2.03 ± 0.36	1.95 ± 0.34
Eosinophils (10^3^/*μ*L)	0.06 ± 0.01	0.11 ± 0.02	0.08 ± 0.019	0.12 ± 0.03	0.04 ± 0.02	0.02 ± 0.01
Platelets (10^3^/*μ*L)	504.80 ± 133.92	878.30 ± 70.84	737.33 ± 72.40	794.50 ± 89.33	649.83 ± 113.40	1108.75 ± 99.56^a^
Red blood cells (10^6^/*μ*L)	10.11 ± 1.01	8.39 ± 0.60	8.22 ± 0.51	7.39 ± 0.73	8.27 ± 1.30	8.54 ± 0.54
Hemoglobin (g/dL)	17.06 ± 1.74	14.80 ± 0.64	14.55 ± 0.59	14.00 ± 0.78	15.81 ± 1.16	14.06 ± 1.18
Hematocrit (%)	71.92 ± 7.50	61.31 ± 3.50	59.28 ± 2.71	55.06 ± 4.44	60.40 ± 8.73	59.70 ± 4.96
Mean cell volume (fL)	71.18 ± 0.97	73.60 ± 1.98	72.60 ± 1.88	75.61 ± 2.79	74.78 ± 2.39	71.33 ± 2.10
Mean cell hemoglobin (pg)	16.86 ± 0.24	17.98 ± 1.25	17.91 ± 0.96	20.15 ± 2.93	23.26 ± 5.95	16.80 ± 0.05
Mean cell hemoglobin concentration (g/dL)	23.76 ± 0.23	24.33 ± 1.05	24.61 ± 0.65	26.16 ± 2.59	30.2 ± 6.36	23.56 ± 0.65

Values are expressed as mean ± SEM, *n* = 6. Nor = administered distilled water; Neg = ART only; Pos = ART + silymarin; ART + PNS 200 mg; ART + PNS 400 mg; ART + PNS 800 mg. ^a^*p* < 0.05 significant difference from the normal control group.

**Table 2:** Effect of 17 mg/kg TLE regimen and PNS extract on the hepatic, renal, and lipid functions.

	Normal	Negative group	Positive control	ART + PNS200	ART + PNS400	ART + PNS800
ALT (U/L)	24.28 ± 5.88	26.32 ± 8.35	25.72 ± 7.26	90.82 ± 27.53^a^	29.89 ± 6.12	30.62 ± 5.47
AST (U/L)	75.42 ± 6.68	84.87 ± 6.37	78.13 ± 6.12	71.75 ± 5.17	55.48 ± 3.56^b^	68.35 ± 7.75
ALP (U/L)	7.75 ± 0.99	4.67 ± 0.50^c^	2.97 ± 0.76^d^	1.98 ± 0.33^e^	2.06 ± 0.98^f^	0.82 ± 0.01^g^
CR (mg/dL)	0.65 ± 0.11	0.50 ± 0.06	0.57 ± 0.05	0.59 ± 0.11	0.67 ± 0.09	0.67 ± 0.07
TG (mg/dL)	54.51 ± 2.58	68.38 ± 10.52^h^	45.95 ± 2.05^i^	49.84 ± 2.11^j^	46.26 ± 5.55^k^	44.39 ± 1.81^l^
TC (mg/dL)	95.48 ± 2.10	93.38 ± 4.37	104.96 ± 5.31	93.98 ± 2.04	85.92 ± 7.01	100.72 ± 2.41
TP (mg/dL)	0.61 ± 0.13	1.42 ± 0.18^m^	0.70 ± 0.14^n^	1.31 ± 0.13	0.91 ± 0.16	1.13 ± 0.04

Values are expressed as mean ± SEM, *n* = 6. Nor = administered distilled water; Neg = ART only; Pos = ART + silymarin; ART + PNS 200 mg; ART + PNS400 mg; ART + PNS 800 mg. ^a,b,c,d,e,f,g,h,I,j,k,l,m,n^*p* < 0.05 significant difference from the normal control group. ALT = alanine aminotransferase; AST = aspartate aminotransferase; ALP = alkaline phosphatase; CR = creatinine; TG = triglycerides; TC = total cholesterol; TP = total proteins.

## Data Availability

The datasets used and analyzed during the study are available without any restriction.

## Abbreviations

ABTS: 2,2'-Azinobis-(3-ethylbenzothiazolin-6-sulfonic acid)
AIDS: Acquired immune deficiency syndrome
ALP: Alkaline phosphatase
ALT: Alanine aminotransferase
ART: Antiretroviral therapy
AST: Aspartate aminotransferase
CAM: Complementary and alternative medicine
CC_50_: Half maximal cytotoxic concentration
CRPMT: Centre for Research on Medicinal Plants and Traditional Medicine
CYP: Cytochrome P450
DILI: Drug-induced liver injury
DPPH: 1,1-Diphenyl-2-picrylhydrazyl
FRAP: Ferric reducing antioxidant power
GSH: Reduced glutathione
HAART: Highly active antiretroviral therapy
HBV: Hepatitis B virus
HIV: Human immune deficiency virus
IC_50_: Half maximal inhibitory concentration
IMPM: Institute for Medical Research and Medicinal Plant Studies
MINRESI: Ministry of Scientific Research and Innovation
NNRTI: Nonnucleos(t)ide reverse transcriptase inhibitor
NRTI: Nucleos(t)ide reverse transcriptase inhibitor
PIs: Protease inhibitors
PNS: *Piper nigrum* stem
RNA: Ribonucleic acid
ROS: Reactive oxygen species
SOD: Superoxide dismutase
SSA: Sub-Saharan Africa
TB: Tuberculosis
TC: Total cholesterol
TG: Triglycerides
TLE: Tenofovir 300 mg/lamivudine 300 mg/efavirenz 400 mg
WHO: World Health Organization.
